# Sol-Gel Derived Mg-Based Ceramic Scaffolds Doped with Zinc or Copper Ions: Preliminary Results on Their Synthesis, Characterization, and Biocompatibility

**DOI:** 10.1155/2016/3858301

**Published:** 2016-02-14

**Authors:** Georgios S. Theodorou, Eleana Kontonasaki, Anna Theocharidou, Athina Bakopoulou, Maria Bousnaki, Christina Hadjichristou, Eleni Papachristou, Lambrini Papadopoulou, Nikolaos A. Kantiranis, Konstantinos Chrissafis, Konstantinos M. Paraskevopoulos, Petros T. Koidis

**Affiliations:** ^1^Department of Physics, Aristotle University of Thessaloniki, 54124 Thessaloniki, Greece; ^2^Dentistry Department, Laboratory of Fixed Prosthesis and Implant Prosthodontics, Aristotle University of Thessaloniki, 54124 Thessaloniki, Greece; ^3^Department of Geology, Aristotle University of Thessaloniki, 54124 Thessaloniki, Greece

## Abstract

Glass-ceramic scaffolds containing Mg have shown recently the potential to enhance the proliferation, differentiation, and biomineralization of stem cells in vitro, property that makes them promising candidates for dental tissue regeneration. An additional property of a scaffold aimed at dental tissue regeneration is to protect the regeneration process against oral bacteria penetration. In this respect, novel bioactive scaffolds containing Mg^2+^ and Cu^2+^ or Zn^2+^, ions known for their antimicrobial properties, were synthesized by the foam replica technique and tested regarding their bioactive response in SBF, mechanical properties, degradation, and porosity. Finally their ability to support the attachment and long-term proliferation of Dental Pulp Stem Cells (DPSCs) was also evaluated. The results showed that conversely to their bioactive response in SBF solution, Zn-doped scaffolds proved to respond adequately regarding their mechanical strength and to be efficient regarding their biological response, in comparison to Cu-doped scaffolds, which makes them promising candidates for targeted dental stem cell odontogenic differentiation and calcified dental tissue engineering.

## 1. Introduction

Research on “engineered tissues” is remarkably growing in recent years, as there is an increased demand of many clinical specialties for biomaterials able not only to substitute the lost or destroyed tissues but also to provide an environment that could induce its own regeneration. Extended research has started to emerge in the field of dental tissue regeneration based on the use of stem cells in combination with various scaffolds and relevant growth and differentiation factors which make the classical tissue engineering triad [1]. In literature, various types of biomaterial scaffolds have been developed as ECM analogs capable of supporting cell attachment, proliferation, and differentiation and, ultimately, forming new engineered tissues or organs [2, 3]. Although a wide range of biomaterials have been proposed for this purpose (ceramics, natural or synthetic polymers, etc.), to date, glass, glass-ceramic, and ceramic scaffolds present important advantages compared to polymeric scaffolds, such as porous structure and chemical texture that promotes mesenchymal cells differentiation and mineralization of the extracellular matrix, lack of toxic byproducts, and the formation of dentinal tubule-like structures [4–7]. Furthermore, ceramic scaffolds can be used as carriers of growth factors and angiogenetic agents, drugs, and cell differentiation products [8, 9]. Calcium-phosphate (*β*-TCP) or hydroxyapatite (HA) scaffolds are the most investigated compositions for dental tissues regeneration due to their chemical resemblance to the mineral component of natural dentin in mammals. However, other ceramic compositions may be more beneficial in triggering dental tissue formation, although research in this area is limited. Mg-doped phosphate glasses and ceramics have been shown to enhance the bioactivity of the scaffolds related to osteogenesis [10]. However, to date, the effect of Mg ions on dentin regeneration is largely unknown, although the use of Mg-containing ceramic scaffolds for dentin regeneration seems a reasonable concept due to the increased amount of Mg contained in dentin [11]. Based on the fact that magnesium plays a fundamental role in cellular processes [12, 13] and skeletal metabolism [14, 15], Mg-containing glass-ceramics with high porosity, suitable degradability, and bioactivity have been only recently proposed for dental tissue regeneration [16]. Huang et al. [17] compared akermanite (Ca_2_Mg(Si_2_O_7_)) and *β*-tricalcium phosphate (*β*-TCP) in their ability to induce differentiation of human mesenchymal stem cells (MSCs), showing that the release of Si and Mg significantly facilitated stem cell proliferation and differentiation. Furthermore, Qu et al. [18] reported that the sustained release of Mg ions from magnesium-containing nanostructured hybrid scaffolds significantly enhanced the proliferation, differentiation, and biomineralization of human DPSCs in vitro.

New studies over the introduction of various metallic ions when synthesizing bioactive glasses report that when used in small amounts, they could be beneficiary [19], since by tailoring the composition and ionic dissolution process of bioactive glasses, the stimulation of specific cell behavior may be achieved. The addition of Cu ions has been proposed to show beneficial effects on angiogenesis [6] and to induce an increase in differentiation of MSCs [7] whereas the addition of Zn ion shows anti-inflammatory effects and stimulates bone formation in vitro by activating protein synthesis in osteoblasts [8, 9]. Recently biomaterials with antibacterial properties have been suggested in dental tissues engineering for the creation of a bacteria-free environment while healing and regenerating the defect area. This is particularly important for regenerating dental tissues which are prone to bacterial invasion from the oral cavity. The synthesis of three-dimensional porous scaffolds with interconnected porous structure, able to function as temporary 3D templates for cell attachment, proliferation, and differentiation when in contact under controlled environment and capable of releasing ions with antimicrobial or cariostatic properties [12], could constitute a suitable inductive carrier that could enhance dentin regeneration and induce the optimal formation of new dentin matrix. Although the last few years Mg, Zn, and Cu ions have shown promising results as additives or dopants to bioceramic scaffolds, the effect of their simultaneous presence in quaternary systems of SiO_2_-CaO-MgO-CuO or SiO_2_-CaO-MgO-ZnO has not been investigated, to the best of the authors' knowledge. Consequently, the aim of this work was to synthesize Mg-based glass-ceramic scaffolds with incorporated Zn/Cu ions and to investigate their physical, mechanical, and biological properties.

## 2. Materials and Methods

### 2.1. Scaffold Fabrication

Mg-based scaffolds of different composition as indicated in Table 1 were synthesized. Polyurethane (PU) foam was used as a sacrificial template in order to produce 3D porous scaffolds. The foam was cut into pieces of 10 × 10 × 5 mm and used for the fabrication of the bioactive scaffolds through the foam replica technique as described by Chen et al. [20] while it was immersed in sol-gel. The sol-gel solution was prepared as described by Goudouri et al. [16]. Briefly, TEOS was added in the mixture of ultrapure H_2_O and HNO_3_ (2 N) and stirred—for approximately 30 min—until partial hydrolysis of TEOS occurred. Calcium nitrate tetrahydrate (Ca(NO_3_)_2_·4H_2_O), magnesium nitrate hexahydrate (Mg(NO_3_)_2_·6H_2_O), and zinc nitrate hexahydrate (Zn(NO_3_)_2_·6H_2_O) or cupric nitrate hemipentahydrate (Cu(NO_3_)_2_·2.5H_2_O) were added to the mixture allowing 50 min for the hydrolysis reaction to complete at 60°C. After the immersion of the foam in the sol-gel and mechanical stirring for 5 min, the samples (green bodies) were retrieved from the sol-gel and squeezed in order to remove the excess of sol from the pores and then left to dry out for at least 12 h. The thickness of the bioactive glass on the green bodies was adjusted by pouring droplets of sol-gel. The excess was removed after centrifuging the green bodies.

In order to understand the structural changes upon heating of the bioactive glasses, as well as their mass loss percentage, the Thermogravimetric (TG) and Differential Scanning Calorimetry (DSC) curves were received with heating rate of 10°C/min from room temperature to 1400°C, under nitrogen atmosphere. According to TG-DSC results, the synthesized bioactive scaffolds were sintered at 890°C (Zn-based) and 866°C (Cu-based) with 2 h annealing.

### 2.2. Characterization

Fourier Transform Infrared Spectroscopy (FTIR) and X-ray diffraction analysis (XRD) were used in order to examine thoroughly the scaffold's crystal structure. For FTIR measurements a Perkin-Elmer Spectrometer Spectrum 1000 in MIR region was used to determine the chemical composition of the fabricated scaffolds. Representative scaffolds from both groups were ground into powder and pellets with powder to KBr ratio of 1 : 100 were fabricated under pressure (7 tons). For the XRD analysis a Philips (PW1710) diffractometer with Ni-filtered CuKa wave radiation was used. The scaffolds were crushed and ground into powder for XRD analysis. Archimedes method was used for the determination of the scaffold porosity, *p*, as indicated by the equation

(1) $$\begin{array}{c} p=\frac{V_{pores}}{V_{bulk}}, \end{array}$$

where *V*_bulk_ was calculated by the mass and dimensions of the scaffolds. The morphology and microstructure of the scaffolds was monitored by the use of scanning electron microscopy with associated energy dispersive spectroscopic analysis (SEM-EDS).

### 2.3. Compressive Strength Evaluation

The mechanical properties of the synthesized scaffolds were tested by an Instron 3344 loading apparatus in compression at a crosshead speed of 0.5 mm/min. Ten prismatic samples (five from each group) with dimensions 8 × 8 × 4 mm were tested. The compressive load was applied until 1 mm (12.5%) compressive strain was achieved in the 8 mm dimension (height). Further loading applied during pilot experiments resulted in off-axis loading and thus the received stress values were not considered as valid. The highest stress values included in the 1 mm stress-strain curve were recorded and mean values with standard deviations were determined.

### 2.4. In Vitro Degradation

Degradation test was performed according to the ISO 10993-14: 2009 (extreme and simulation solution tests). More specifically, 12 scaffolds of each group were tested. Mass calculation was performed with an electronic balance (Kern ABS) with an accuracy of 0.0001 mg and the specific mass of each scaffold was recorded as the difference between the mass of the container with and without the scaffold. For the simulation solution test each container was filled with 100 mL of freshly prepared TRIS-HCL buffer, with pH 7.4 ± 0.1 at 37 ± 1°C, while for the extreme solution test each container was filled with 10 mL of the buffered citric acid solution, with pH 3.0 ± 0.2 at 37 ± 1°C. Then, all containers were placed in a controlled-temperature environment at 37 ± 0.5°C, for 120 h. The containers were agitated at 2 Hz with circular movement. After 120 h the containers with scaffolds were allowed to cool at room temperature. Remnants of scaffolds were removed under filtration. Reweighted filter paper was used for filtration. Remnants were rinsed and filtrated three times with small amounts of water grade 2. Then, filter paper and scaffold's remnants were dried in an oven overnight at 100 ± 2°C. Drying procedure was continued until mass changes less than 0,1% were recorded. The difference between the mass of the filter paper with and without the remnants was the actual mass of the nondegraded scaffold. Finally, the difference between the initial recorded mass of the scaffolds and the mass of the nondegraded scaffold remnants was recorded as the mass of the degraded scaffold. The % weight loss was determined with the following equation:

(2) $$\begin{array}{c} Weight\;\;loss\;\left(\;\%\;\right)=\left[\;\frac{\left(W_{o}-W_{t}\right)}{W_{o}}\;\right]\times 100. \end{array}$$

### 2.5. Apatite Forming Ability in SBF

The scaffolds were placed in sterilized reagent bottles and submerged in SBF solution with mass to solution ratio adjusted at 1.5 mg/mL [21]. Then they were placed in an incubator (Incucell 55) at 37°C under renewal conditions for various times after immersion (6 h, 24 h, and then after every 48 h) [22]. Finally the specimens were removed from the SBF at each time point (after 10 and 21 days of immersion), washed with distilled water, and dried at room temperature.

### 2.6. Evaluation of Cell Viability/Proliferation and Cell Attachment/Morphology of Dental Pulp Stem Cells (DPSCs) Seeded into the Biomimetic Scaffolds

DPSC cultures were established from third molars of young healthy donors aged 16–18 years and extensively characterized for several stem cell markers, as previously published by our group [23]. The collection of the samples was performed according to the guidelines of the Institutional Review Board and the parents of all donors signed an informed consent form. For the establishment of cell cultures the enzymatic dissociation method was used [24]. Briefly, teeth were disinfected and cut around the cementum-enamel junction to expose the pulp chamber. The tissue was minced into small segments and digested in a solution of 3 mg/mL collagenase type I and 4 mg/mL dispase II (Invitrogen, Karlsruhe, Germany) for 1 h at 37°C. Single cell suspensions were obtained by passing the cells through a 70 *μ*m cell strainer (BD Biosciences, Heidelberg, Germany). Cells were expanded with *α*-MEM (Minimum Essential Media) culture medium (Invitrogen), supplemented with 15% FBS (EU-tested, Invitrogen), 100 mM L-ascorbic acid phosphate (Sigma-Aldrich, Taufkirchen, Germany), 100 units/mL penicillin, 100 mg/mL streptomycin, and 0.25 mg/mL Amphotericin B (all from Invitrogen) (Complete Culture Medium (CCM)), and incubated at 37°C in 5% CO_2_. Cultured DPSCs in passage numbers from 3 to 6 were used for all experiments.

To analyze cell viability/proliferation the MTT assay was used. Scaffolds were first preimmersed into CCM for 30 min at 37°C and 5% CO_2_ atmosphere in an incubator in order to adjust pH and create a more biomimetic microenvironment before cell seeding. Afterwards, the medium was removed and DPSCs were spotted at low volume (100 *μ*L) into the scaffolds at 5 × 10^5^ cells/scaffold in 48 well-plates and allowed to attach first for 45 min before being fully covered with 500 *μ*L CCM. Cell viability/proliferation was evaluated after 1, 3, 7, and 14 days (d) by the MTT assay (*n* = 4). Medium change was performed every 3 days during the entire experimental period. At the end of each time point 50 *μ*L of MTT (5 mg/mL in PBS) was added in each well and scaffolds/cell constructs were incubated for 3 h at 37°C and 5% CO_2_. After this period, the medium containing the MTT solution was discarded, the scaffold/cell constructs were washed with PBS, and the insoluble formazan was dissolved with DMSO overnight at 37°C. The absorbance was measured against blank (DMSO) at a wavelength of 545 nm and a reference filter of 630 nm by a microplate reader (Epock, Biotek, Biotek instruments, Inc, Vermont, USA). As controls, scaffolds (CuA2 and ZnA2) without cells were incubated under the same conditions and the optical density values were subtracted from values obtained by the corresponding scaffold/cell constructs. Finally, OD values were normalized to those of control DPSCs cultures beginning with the same cell number (5 × 10^5^ cells/well) and the final results were expressed as % percentage of control.

In order to evaluate cell attachment and morphology of DPSCs seeded into the biomimetic scaffolds, samples of scaffold/cell constructs were processed for scanning electron microscopy (SEM). Cells were seeded into the biomimetic scaffolds, as described for the MTT assay. After 3, 7, and 14 d, the scaffold/cell constructs were washed twice with PBS and fixed with 3% glutaraldehyde (in 0.1 M sodium cacodylate, pH 7.4, containing 0.1 M sucrose). The specimens were subsequently dehydrated in a series of increasing concentrations of ethanol and hexamethyldisilazane. For SEM analysis they were carbon-coated and observed with a Jeol (Japan) electronic microscope at 20 kV.

## 3. Results and Discussion

### 3.1. TG – DSC Analysis

**Figure 1:**
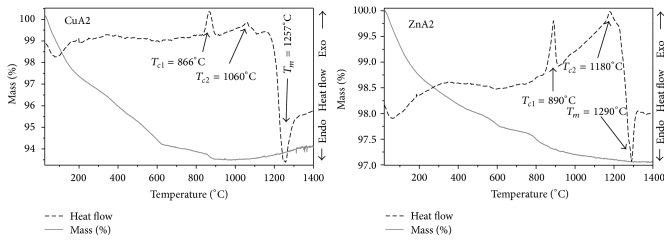
TG and DSC curves of CuA2 and ZnA2 glass powders.

Thermal analysis of bioactive glasses can efficiently determine mass variations and thermal content changes as a function of temperature. Therefore, it is critically essential to determine these variations and changes for every material, which is going to receive heat treatment, in order to be able to predict its behavior at high temperatures. As already mentioned, ZnA2 scaffolds were sintered at 890°C and CuA2 scaffolds at 866°C, with 2 h annealing. Those temperatures were extracted from DSC curves (Figure 1, red lines) for each bioactive glass and represent exothermic peaks (*T*_*c*_). The temperature at these peaks corresponds to the crystallization of the samples, while additional exothermic peaks are observed at higher temperatures (1060°C for CuA2 and 1180°C for ZnA2). Endothermic peaks assigned to the melting point (*T*_*m*_) of each sample are observed at 1257°C for the CuA2 and 1290°C for the ZnA2 glasses. In this study, sintering temperatures were chosen at the first exothermic peak of each glass, in order to produce scaffolds with improved mechanical properties, as the crystallization of a glass provides a mechanically enhanced system without necessarily impairing the bioactive response of the glass-ceramic [25].

The TG curves (Figure 1, blue lines) of both samples indicate that the mass variations were insignificant, being under 8% for both of them. Mass loss for both bioactive glasses takes place under 600°C and is caused because of the H_2_O, CO, and CO_2_ release from the samples, which were entrapped inside during the synthesis process.

### 3.2. Characterization of the Fabricated Scaffolds

**Figure 2:**
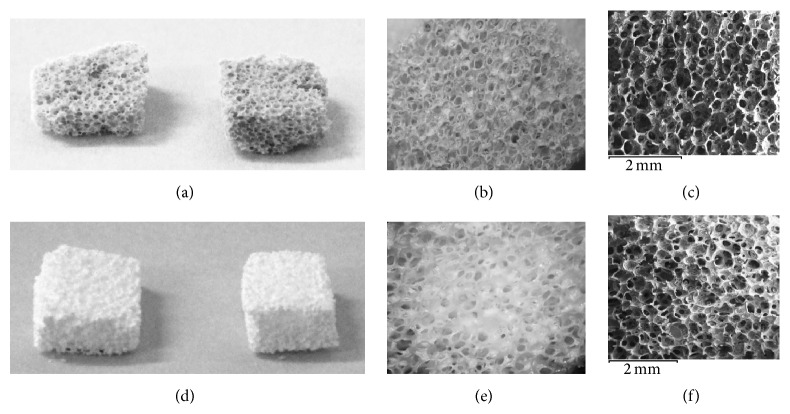
Digital camera photographs (a, d), light microscope images of different magnifications (b, e), and SEM microphotographs (c, f) of the glass-ceramic scaffolds (a, b, c: CuA2, d, e, f: ZnA2).

The glass-ceramic scaffolds were successfully fabricated via the foam replica technique. Bioactive scaffolds in order to be applied for the development of calcified tissue should be able to favor cell penetration, vascularization, and nutrient and metabolic waste transportation [26, 27]. To achieve such a goal scaffolds should exhibit interconnected porous structure with pore sizes between 300 and 500 *μ*m [18, 28]. The porous structure and morphology of the bioactive glass-ceramic scaffolds are shown in Figure 2.

SEM microphotographs revealed pore size of approximately 200–400 *μ*m and interconnected pore structure. The ZnA2 and CuA2 scaffolds presented a mean porosity of 84% and 74%, respectively. A primary goal of dental tissue regeneration is the development of suitable scaffolding materials that could support dental stem cells attachment and proliferation. Scaffolds of similar porosity and interconnectivity as those of the scaffolds fabricated in this study have been shown to support the attachment and proliferation of human Dental Pulp Stem Cells [18, 28].

**Figure 3:**
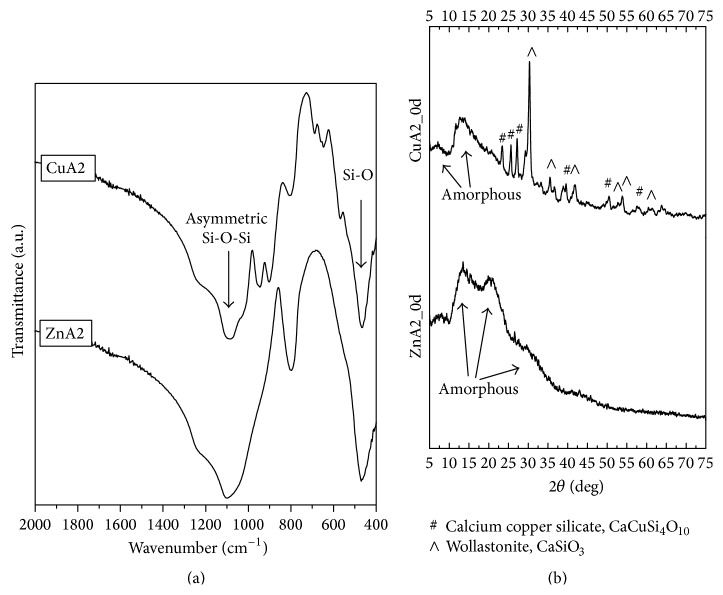
(a) FTIR spectra and (b) XRD patterns of ZnA2 and CuA2 glass-ceramic scaffolds.

**Figure 4:**
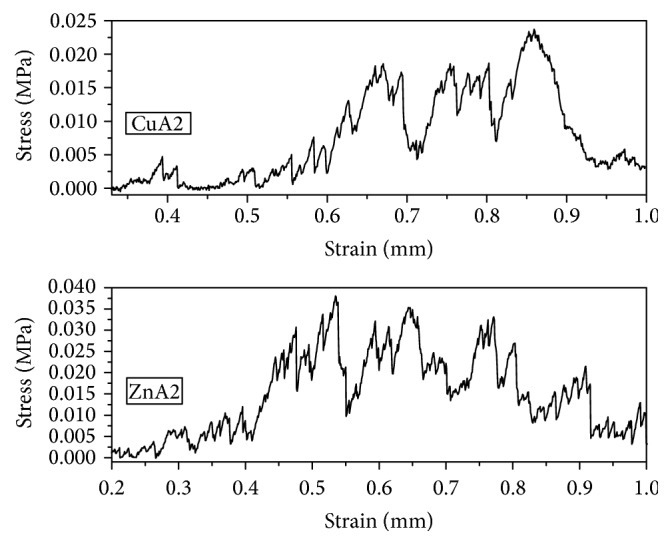
Indicative stress (MPa)-strain (mm) curves for each group of glass-ceramic scaffolds. The compressive load was applied until 1 mm (12.5%) compressive strain was achieved along the 8 mm dimension (height).

The FTIR spectra of the fabricated scaffolds are shown in Figure 3. FTIR spectra of both ZnA2 and CuA2 glass-ceramic scaffolds present the characteristic peaks of silicate glasses shown by a broad peak at 900–1200 cm^−1^ and the peak at ~470 cm^−1^ [29]. In addition, the spectra of the ZnA2 reveal the presence of a strong peak at 796 cm^−1^ indicating the existence of bridging oxygen, which are connected with the inability of a glass-ceramic material to exhibit bioactive behavior [30, 31]. This peak—though present—is not so intense in the spectra of the CuA2 scaffolds. For CuA2 scaffolds, the FTIR peaks at 646 cm^−1^, 690 cm^−1^, 902 cm^−1^, and 946 cm^−1^ were attributed to wollastonite (CaSiO_3_) [32]. XRD patterns (Figure 4) revealed that ZnA2 scaffolds consist mainly of an amorphous phase. On the other hand, XRD patterns of CuA2 glass-ceramic scaffolds indicate the existence of wollastonite (approximate percentage 40%wt), while 10%wt of calcium copper silicate (CaCuSi_4_O_10_) was also detected. These findings confirmed the FTIR results.

### 3.3. Compressive Strength

The mechanical strength of both ZnA2 and CuA2 scaffolds under uniaxial compression stress was proven, as expected, rather low but in the range of values attained for ceramic scaffolds noncoated with gelatin or other polymeric materials [33, 34]. More specific, ZnA2 glass-ceramic scaffolds presented a mean compressive strength at 0.10 (±0.06) MPa and CuA2 glass-ceramic scaffolds a mean compressive strength of 0.02 (±0.007) MPa. As it is shown from the typical stress-strain curves presented in Figure 4, a continuous section with peaks after isolating linear elastic regions and valleys, corresponding to the brittle crushing of the struts, was the dominant mode of fracture, as has been observed for brittle ceramic porous scaffolds [34, 35]. Further improvements of the mechanical properties of the scaffolds are necessary for the maintenance of their structural integrity so as to allow time for the new calcified tissue to grow. The capability of improving the mechanical properties of ceramic scaffolds has been demonstrated in several composite polymer-ceramic formulations [36–39]. It is highly possible that coating these scaffolds with gelatin or alginate hydrogel could significantly improve their mechanical behavior and this is a subject of future research.

### 3.4. In Vitro Degradation

**Figure 5:**
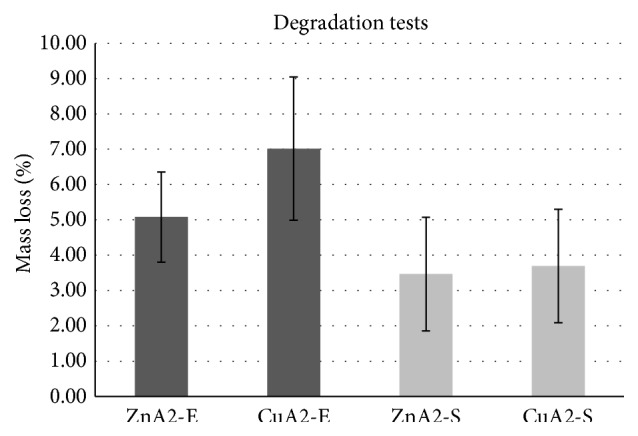
Results of degradation tests. Zn-S: ZnA2 conventional test, Cu-S: CuA2 conventional test, Zn-E: ZnA2 extreme test, and Cu-E: CuA2 extreme test.

Results of degradation tests are presented in Figure 5. A mean degradation rate of 3.5% (ZnA2)-3.7% (CuA2) was recorded for the simulation test in Tris Buffer solution after 120 h immersion, while extreme test in citric acid solution revealed slightly higher degradation rate for the same time period (5% for ZnA2, 7% for CuA2). Both tests resulted in low solubility values. As it was expected the recorded degradation values were higher for the extreme test in comparison to the simulation test for both ceramic scaffolds (ZnA2 and CuA2). Moreover, ceramic scaffolds of ZnA2 presented lower degradation values in extreme degradation test, in comparison with CuA2, while the two ceramic scaffolds presented almost equal degradation values in simulation degradation test. The higher degradation rate of the CuA2 compared to the ZnA2 scaffolds can be explained by the presence of wollastonite in the CuA2 scaffolds, as it has been found that the increase of wollastonite percentage rapidly increases the mass loss of composite poly(3-hydroxybutyrate-*co*-3-hydroxyvalerate) (PHBV)/wollastonite scaffolds [40]. This increased weight loss may be attributed to the dissolution of wollastonite when immersed in aqueous solution, the release of alkaline ions, and the subsequent destruction of the three-dimensional structure of the scaffolds. The release of alkaline ions of bioactive glasses is one of the basic mechanisms of apatite formation, as it leads to the formation of a high surface area of hydrated silica and finally to the crystallization of apatite through precipitation of P and Ca from the surrounding environment [41]. The increased degradation rate may be the reason for the bioactive behavior of CuA2 despite its lower porosity, while the lower degradation of ZnA2 that can be assigned to the presence of nonbridging oxygen as found with FTIR explains its inability for in vitro apatite formation.

Degradation of scaffolds is necessary during calcified tissue formation, as scaffolds are networks that assist initial cell attachment and proliferation but have to degrade simultaneously with the new tissue formation. Similar degradation rate values with those of the present study have been recorded for bioceramic scaffolds in literature [42, 43], although usually degradation rate of bioceramics is evaluated by measuring mass loss after immersion in solutions like SBF [44] or PBS [40] due to their resemblance with physiological body fluids. In this study degradation of ceramic scaffolds was evaluated according to ISO 10993-14: 2009 which is more appropriate for testing materials in contact with fluids of different pH. The simulation test is a mild, common test used to evaluate the degradation rate of most ceramic materials under physiological pH and temperature similar to physiological body fluids, while the extreme test is related to the more aggressive environment to which a material can be exposed to in the oral cavity due to low pH. Remarkable variation in degradation values was recorded among the scaffolds of both groups. Slight differences in scaffold's porosity or structure through the fabrication process could explain such variation [42, 45]. Greater degradation values were reported in literature, only for significantly longer immersion period (14–28 days) [43, 46]. Lower degradation values were recorded after 3 days of immersion in Tris Buffer for wollastonite/tricalcium phosphate macroporous scaffolds, with significantly lower porosity (50%) [45]. The degradation rate of Ca-P bioceramics is influenced by several parameters such as the sintering process, microstructure, crystallinity, and porosity [47]. The porosity plays a dominant role in the degradation of bioceramics as it is related to high specific surface area [48]. However the results of this study indicate that other mechanisms rather than porosity may be more crucial in determining the degradation of the scaffolds, such as composition and crystalline structure.

### 3.5. Bioactivity Evaluation

**Figure 6:**
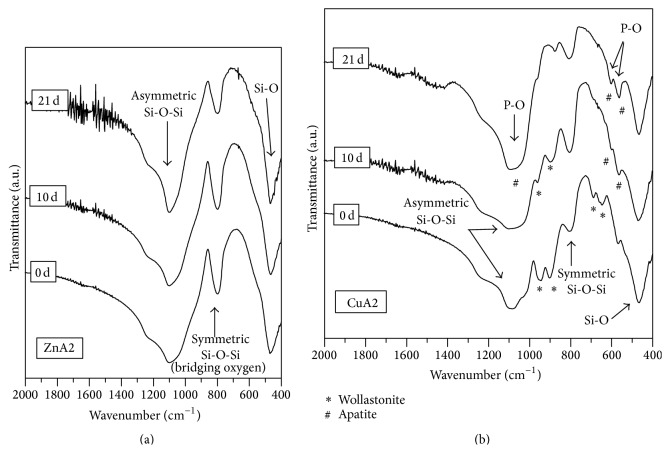
FTIR spectra of (a) ZnA2 and (b) CuA2 glass-ceramic scaffolds before and after immersion in SBF solution for 10 and 21 days.

FTIR spectra of ZnA2 glass-ceramic scaffolds could not reveal any differentiation in their chemical composition even after 21 days of immersion in SBF solution, as shown in Figure 6(a). This result may be attributed to the presence of bridging oxygen as shown by the strong peak at 796 cm^−1^, as already mentioned.

On the contrary, CuA2 bioactive scaffolds presented bioactive behavior according to FTIR spectra (Figure 6(b)). More specific, FTIR spectra, after 10 days of immersion, revealed the formation of a weak double peak at 587 cm^−1^ and 603 cm^−1^, which is attributed to the vibration of the P-O bond of the phosphate group. The high amount of wollastonite crystallized on the initial material could explain the delayed formation of apatite on the surface of CuA2 glass-ceramic scaffolds. This double peak is known to be associated with apatite formation. After 21 days of soaking in SBF solution, a stronger double peak at 587 cm^−1^ and 603 cm^−1^ was formed. Additionally, at the same immersion time, the broad peak at 900–1200 cm^−1^ shifted towards ~1100 cm^−1^ and became less wide. Therefore, after 21 days of soaking in SBF there is a strong indication of the formation of apatite on the surface of the glass-ceramic scaffolds of the CuA2 group.

**Figure 7:**
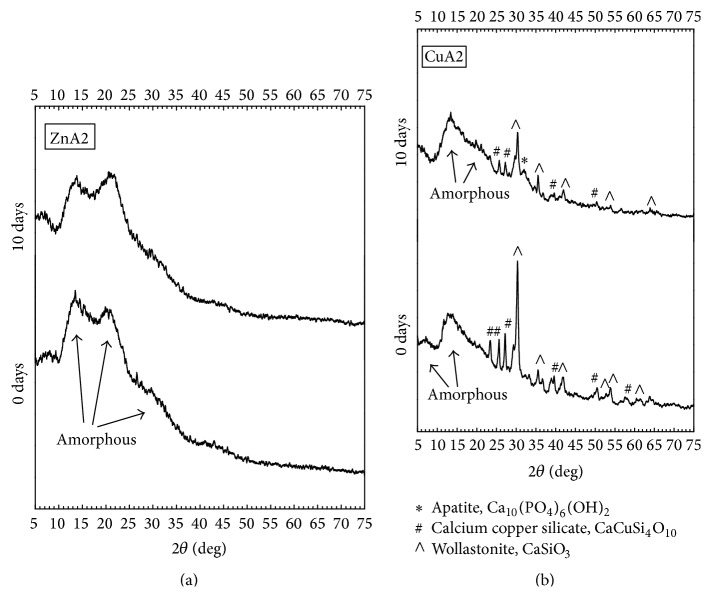
XRD spectra of (a) ZnA2 and (b) CuA2 glass-ceramic scaffolds before and after immersion in SBF solution for 10 days.

These findings are in accordance with XRD patterns (Figure 7) for both groups of glass-ceramic scaffolds. Namely, ZnA2 samples did not show any compositional differentiation after 10 days of immersion in SBF solution, whereas CuA2 patterns revealed a peak corresponding to apatite after 10 days of soaking.

The effect of zinc incorporation on the structure of various bioactive glasses has resulted in different results concerning bioactivity depending on the microstructure and physicochemical properties of Zn-doped glasses. Although the acellular formation of calcium phosphate layer on the surface of bioactive silicate glasses doped with Zn have been shown to occur after soaking in biological fluids [49, 50], other studies have shown that Zn content reduces the overall leaching activity of the glass inhibiting the formation of the HCA layer on its surface [51]. Haimi et al. [52] reported a delayed formation of HCA which was related to the slower degradation profile of the Zn-doped bioactive glasses, in accordance with the results of this study. On the other hand, Cu^2+^-doped 45S5 BG scaffolds exhibit high apatite forming ability, as proven by the rapid formation of a carbonated HA layer on their surface (3 days in SBF) [53]. Hoppe et al. [53] reported that Cu^2+^ addition (up to 2.5wt% CuO) had no effect on the reactivity of the undoped BG, as measured through immersion in SBF. Goudouri et al. [16] fabricated sol-gel Mg-based scaffolds with the foam replica technique and reported apatite formation on scaffolds sintered at 1350°C after 9 days in SBF. As the authors in the current study used the same starting glass formulation, the incorporation of copper resulted in a slight delay of apatite formation, taking into consideration the lower crystallization temperature of the scaffolds.

### 3.6. Evaluation of Cell Viability/Proliferation and Attachment/Morphology of DPSCs on the Biomimetic Scaffolds

**Figure 8:**
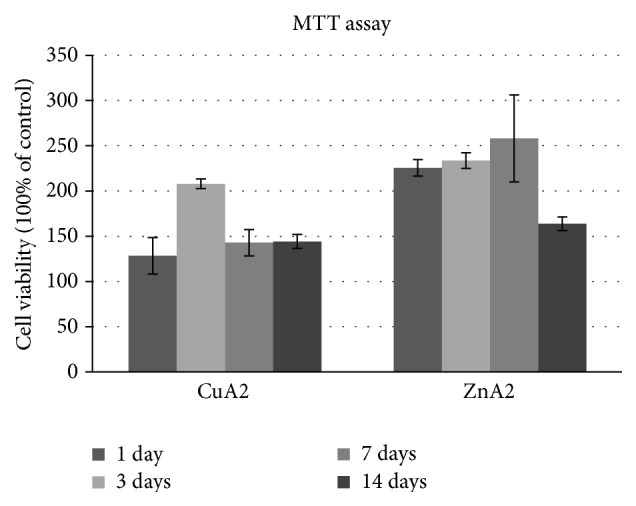
Evaluation of cell viability/proliferation of DPSCs seeded into the CuA2 and ZnA2 bioceramic scaffolds for 1, 3, 7, and 14 days (MTT assay).

**Figure 9:**
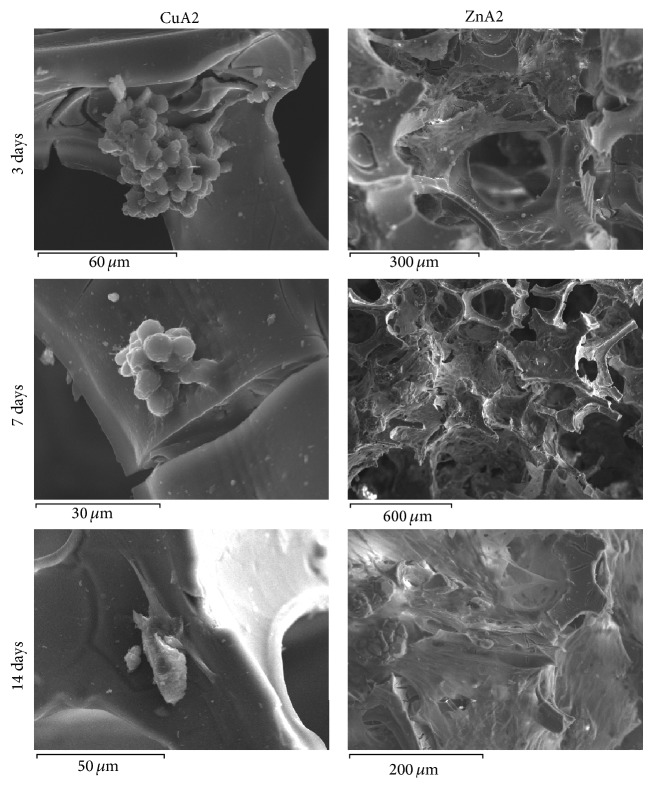
SEM microphotographs of CuA2 and ZnA2 scaffolds seeded with DPSCs after 3, 7, and 14 days.

DPSCs were able to attach and proliferate in both biomimetic scaffolds (Figures 8 and 9). However, both MTT assay and SEM analysis revealed a much better biological behavior of ZnA2 compared to the CuA2 scaffolds. ZnA2 supported a statistically significant higher viability of DPSCs compared to the CuA2 scaffolds at all time points tested (*p* < 0.01). ZnA2 scaffolds showed an increase of cell viability/proliferation up to day 7 and decrease afterwards, which can be explained by the initiation of differentiation of DPSCs inside the biomimetic microenvironment, which is usually accompanied by a cease in proliferation, as already shown in preliminary experiments with real-time PCR analysis and western blotting (data under preparation). On the contrary CuA2 scaffolds showed much lower OD values compared to the ZnA2 scaffolds, with viability/proliferation increasing until day 3 and significantly decreasing afterwards. Whether this inferior biological behavior of CuA2 scaffolds is due to a very high release of cytotoxic concentrations of Cu or any other elements needs further investigation.

The results of the MTT assay were also in accordance with the results obtained by the SEM analysis. Cells grown inside the ZnA2 scaffolds were more densely seeded, with an atractoid, spindle-shaped morphology, indicative of proper attachment and high viability of cells within the scaffold. Cells grown inside the CuA2 scaffolds, on the other hand, were fewer and with a rather rounded morphology, indicative of poor attachment and potential cytotoxicity. Zn-doped sol-gel derived glasses based on 58S have shown higher cellular viabilities than similar Cu-doped glasses, in a recent study by Bejarano et al. [54], although both were cytotoxic compared to undoped control 58S. The enhanced cell behavior recorded in the present study is probably attributed to a more stabilized Zn-derived glass structure that restricted mass glass dissolution and ion release that could exert cytotoxic behavior. Preliminary, unpublished data of the authors suggest that ZnA2 scaffolds combined with DPSCs and growth/morphogenetic factors such as Dentin Matrix Protein, DMP-1, and Bone Morphogenetic Protein, BMP-2, promote odontogenic differentiation and dentin-like tissue formation. These data need further investigation regarding the underlying molecular mechanisms of this biological response.

## 4. Conclusions

Bioactive ceramic scaffolds, with adequate porosity, over 74%, and pore interconnectivity were produced by the foam replica technique. Cu-doped Mg-based scaffolds revealed apatite forming ability after 10 days immersion in SBF, while Zn-doped Mg-based scaffolds failed to develop apatite formation even after 21 days in SBF. Differences in structure are responsible for the different degradation profile, mechanical behavior, and bioactivity of the synthesized scaffolds. Despite failure to develop apatite ZnA2 scaffolds were proved very efficient to provide controlled degradation rate and a biomimetic environment for the long-term attachment and growth of DPSCs (up to 14 days), which makes them very promising for further research on their potential to induce odontogenic differentiation of DPSCs and calcified dental tissue production for targeted dentin regeneration.

## Figures and Tables

**Table 1:** Bioactive scaffold compositions in %wt.

	SiO_2_	CaO	MgO	ZnO	CuO
ZnA2	60	30	7.5	2.5	—
CuA2	60	30	7.5	—	2.5
